# Lanthanide and Ladder-Structured Polysilsesquioxane Composites for Transparent Color Conversion Layers

**DOI:** 10.3390/ma16062537

**Published:** 2023-03-22

**Authors:** Jaehyun Han, Darya Burak, Valeriia Poliukhova, Albert S. Lee, Hoseong Jang, Seungsang Hwang, Kyung-Youl Baek, Joonsoo Han, Byeong-Kwon Ju, So-Hye Cho

**Affiliations:** 1 Materials Architecturing Research Center, Korea Institute of Science and Technology, 5 Hwarang-ro 14-gil, Seongbuk-gu, Seoul 02792, Republic of Korea; 2 Display and Nanosystem Laboratory, College of Engineering, Korea University, Seoul 02841, Republic of Korea; 3 Department of Nanomaterial Science and Engineering, Korea University of Science and Technology, 217 Gajeong-ro, Yuseong-gu, Daejeon 34113, Republic of Korea

**Keywords:** ladder-structured, polysilsesquioxane, lanthanide, color conversion layer, transparent film

## Abstract

Ladder-type polysilsesquioxanes (LPSQs) containing phenyl as a high refractive index unit and cyclic epoxy as a curable unit were found to be excellent candidates for a transparent color conversion layer for displays due to being miscible with organic solvents and amenable to transparent film formation. Therefore, the LPSQs were combined with luminescent lanthanide metals, europium Eu(III), and terbium Tb(III), to fabricate transparent films with various emission colors, including red, orange, yellow, and green. The high luminescence and transmittance properties of the LPSQs–lanthanide composite films after thermal curing were attributed to chelating properties of hydroxyl and polyether side chains of LPSQs to lanthanide ions, as well as a light sensitizing effect of phenyl side chains of the LPSQs. Furthermore, Fourier-transform infrared (FT-IR) and X-ray photoelectron spectroscopy and nanoindentation tests indicated that the addition of the nanoparticles to the LPSQs moderately enhanced the epoxy conversion rate and substantially improved the wear resistance, including hardness, adhesion, and insusceptibility to atmospheric corrosion in a saline environment. Thus, the achieved LPGSG–lanthanide hybrid organic–inorganic material could effectively serve as a color conversion layer for displays.

## 1. Introduction

Inorganic–organic materials such as polysilsesquioxanes (PSQs), mainly synthesized via monomer polymerization of trialkoxysilyl groups, are well-known for their strong cross-linking properties with oxygen to a silicon bond. Their structure can range from the cage- to ladder-like [1,2,3,4,5]. Due to uncontrolled side reactions and difficulties in achieving high molecular weights, ladder polysilsesquioxanes (LPSQs) with a Si–O–Si backbone are challenging to synthesize. Nevertheless, they are greatly desired for their excellent and easily tuned physical and chemical properties. For example, an LPSQ composite with Al_2_O_3_ demonstrated an improved epoxy conversion rate and wear resistance applicable for deformable display windows [6]. Similarly, adding SiO_2_ nanoparticles to LPSQs increased hydrophobicity and thermal resistance for UV-curable coatings [7]. ZnO-incorporated LPSQs were also reported for transparent and luminescent optoelectronic devices owing to their optical transparency feature and superior thermal properties of LPSQs [8]. These advantageous properties, along with the molecular size and high versatility of organic groups bound to the silsesquioxane matrix, can be further adapted or functionalized with lanthanide ions to develop luminescent probes and light emitting devices (LEDs).

Lanthanide-based light emitters acquired substantial interest for their long luminescence lifetimes and large Stokes shifts, making them prospective candidates for the development of optical devices and light conversion materials [9,10]. As a result, numerous application fields utilize lanthanide-based light emitters, including medical diagnostics [11], biological imaging [12], and optical amplifiers [13]. The most commonly utilized lanthanides are Eu^3+^, Tb^3+^, Sm^3+^, and Tm^3+^, with red, green, orange, and blue light emissions in the visible range, respectively. At the same time, Yb^3+^, Nd^3+^, and Er^3+^ are used for their near-infrared (NIR) luminescent properties [14]. To this end, the LPSQs can serve as a useful platform for lanthanide-based hybrid organic–inorganic composites as films in commercial products [15], combining their high optical transmittance, solution processability, organic functionality, and inorganic backbone. However, LPSQs–lanthanide complexes have yet to be studied to the full extent to realize their practical application possibilities.

The typical combinations of hybrid polymer–lanthanide complexes presented in the literature usually have a caged or polyhedral PSQs matrix. For instance, Eu^3+^ doping enhanced red light photoluminescent properties of polyhedral oligomeric silsesquioxanes (POSS), offering solubility and thermal stability for the composite [16]. Moreover, when functionalized with 1,10-phenanthroline groups, POSS with Eu^3+^ enhanced emission intensity and showed high photostability [17]. The Eu^3+^-incorporated POSS hybrid composite was also shown to improve the molecular dispersion properties of lanthanide ions within an inorganic–organic hybrid matrix and provide high photostability, serving as a perfect candidate for luminescent and light-harvesting system development [18]. A Tb^3+^-incorporated bridged PSQs hybrid luminescent film was prepared by sol-gel processing [19], while highly transparent Eu^3+^ and Tb^3+^ luminescent bridged PSQs were obtained by sol-gel processing of a monomer with a large heterocyclic bridged group [20], and under acidic conditions [21]. Architectures similar to Eu^3+^–Tb^3+^-based metal PSQs hybrids with prism-like cage topology exhibited typical lanthanide red and green emissions, where the composite benefited from the lanthanide–polymer coordination and sol-gel chemistries [22]. However, to our knowledge, no studies exist where lanthanides are combined with the LPSQs, known as the most soluble PSQs due to their high molecular weight, exceptional thermal properties, and lack of uncondensed silanol groups [4,23].

Despite the benefits of the lanthanide–LPSQs hybrids, there are significant constraints on attaining emission properties in the film form, including luminescence degradation, which is highly dependent on the synthesis method. Additionally, lanthanide intrinsic drawbacks, such as low thermal and photochemical stability and a tendency to aggregate, further obstruct the full practical implementation of the composite. Furthermore, deposition-based methods such as atomic layer deposition are expensive, whereas conventional physical mixing or blending with polymers results in the loss of inherent emissive properties due to aggregation [24]. Recently, researchers have shown that the dispersion properties of lanthanides could be greatly improved through well-defined organic ligand functionalization in organic polymers of a controlled architecture, such as dendrimers [25] or metal oxide-based coordination of lanthanide ions [26]. Common strategies in previous studies included the use of materials with high optical transmittance and low UV absorption, such as poly(methyl methacrylate) (PMMA) or silica, which allowed to maintain lanthanide emissive properties.

In this study, a high molecular weight LPSQ [27], a unique polymeric analog to the commonly known cage-like POSS [28], was synthesized by a facile method. This fully polymeric material exhibited improved solubility and film formation properties compared to the previously reported POSS [4]. In addition, the proposed method allowed the fabrication of lanthanide-based LPSQ composite emitters with precise control over the organic functional group for additional functionality, such as cross-linking [29]. Moreover, the silanol end group interactions with lanthanide ions enhanced dispersion properties, resulting in stable high emission intensity. As a result, highly transparent films with various emission colors, including red, orange, yellow, and green, were precisely controlled by adjusting the Eu^3+^ and Tb^3+^ ratio, where the lanthanide addition significantly contributed to the improved wear resistance of the films. Hence, the lanthanide-incorporated LPSQ-based transparent films with easily controlled emission colors, high hardness, and strong adhesion can be promising candidates for color-conversion displays.

## 2. Materials and Methods

### 2.1. Materials

Phenyltrimethoxysilane (Gelest, 98%, Morrisville, PA, USA) and 3-glycidoxypropyltrimethoxysilane (Gelest, 98%) were distilled over CaH_2_ prior to use. Potassium carbonate (K_2_CO_3_) (Daejung Chemicals, 99.9%, Siheung-si, Republic of Korea) was dried under vacuum at room temperature prior to use. All other solvents were of reagent grade. Tetrahydrofuran (THF) (Daejung Chemicals) was used as a commercial solvent. Terbium(III) nitrate hexahydrate and europium(III) nitrate pentahydrate were purchased from Sigma-Aldrich (St. Louis, MO, USA).

### 2.2. Synthesis of Ladder-Structured Poly(phenyl-co-glycidoxypropyl)silsesquioxane (LPGSQ64)

The synthesis method of LPGSQ64, with a phenyl:glycidoxypropyl ratio of 6:4, followed the procedure previously reported by our group [30]. Briefly, in a 100-mL round bottom flask, potassium carbonate (0.04 g, 0.29 mmol) was dissolved in deionized water (4.8 mL, 0. 267 mol) with THF (8.9 mL) as a co-solvent until completely transparent. To this solution, a separately prepared mixture of phenyltrimethoxysilane (9.52 g, 0.48 mol) and 3-glycidoxypropyltrimethoxysilane (7.95 g, 0.32 mol) was slowly poured under vigorous stirring. The reaction mixture was stirred for 5 days, after which the molecular weight reached a maximum. Next, the resinous white material was dissolved in dichloromethane and extracted with deionized water at least three times after partial evaporation of the volatiles. Finally, the ladder-structured poly(phenyl-co-glycidoxypropyl)silsesquioxane (named hereafter as LPGSQ64) was obtained as a white powder (13.4 g, 97% crude yield) after the collection of the organic layers, dehydration with anhydrous magnesium sulfate, filtering, and evaporation of dichloromethane.

### 2.3. Fabrication of Lanthanide-Based Hybrid Polymer Composite Films

A simple drop-casting technique was employed to fabricate the composite films. First, the LPGSQ64 powder was mixed with a THF in a 1:4 ratio at room temperature. Then, Eu^3+^ and Tb^3+^ precursors were added to the mixture at various weight percentages (1, 3, 5, 7, 10 wt%) followed by sonication for 30 s. After the process, the mixture was drop-cast onto a slide glass. Thin films were fabricated in alphabet-shaped forms, with a carved commercial sticker applied to the glass. The slide glass was first properly cleaned to eliminate contamination, and then a sticker was attached to it; second, a small amount of the solution mixture was carefully drop-cast on an open glass area. After 5–10 min at room temperature, the sticker was removed, and all samples underwent two stages of heat treatment. First, solvent evaporation proceeded at 60 °C for 30 min to eliminate bubbles, and then thermal curing was conducted at 180 °C for various time intervals.

### 2.4. Measurements

The number average molecular weight (Mn) and molecular weight distributions (Mw/Mn) of the polymers were measured by the JASCO PU-2080 plus system (Easton, MD, USA) equipped with a refractive index detector (RI-2031 plus), UV detector (λ = 254 nm, UV-2075 plus), and Viscotek SLS apparatus (Huston, TX, USA) with THF as the mobile phase at 40 °C with a flow rate of 1 mL min^−1^. The measurement of the samples was carried out through four columns (Shodex-GPC KF-802, KF-803, KF-804, KF-805, Munich, Germany). NMR spectra were recorded in CDCl_3_ at 25 °C on a Bruker Avance (1H: 400 MHz, 29Si: 79.5 MHz, Billerica, MA, USA). FT-IR spectra were measured by a Perkin–Elmer FT-IR system (Spectrum-GX, Waltham, MA, USA) using solvent cast films on polished KBr plates. Thermal gravimetric analysis (TGA) was performed a by the TA instrument TGA 2950 under N_2_ at a temperature ramp rate of 10 °C min^−1^. Differential scanning calorimetry was carried out with the TA Instrument DSC Q20-1426 (New Castle, DE, USA) using a heating rate of 10 °C min^−1^ under N_2_ atmosphere. Optical transmittance of luminescent films was obtained by an ultraviolet–visible (UV–Vis) spectrometer (Varian Cary 100, Agilent Technologies, Santa Clara, CA, USA) in the scan range from 200–800 nm at the resolution of 1 nm. A blank holder without any film was used for background transmittance. Photoluminescence (PL) spectra were measured at room temperature using a Hitachi F-7000 spectrophotometer (Ibaraki, Japan) equipped with a xenon discharge lamp. All samples were measured with a cutoff filter of <420 nm. The FT-IR spectra were acquired in the scan range from 5000–500 cm^−1^ using an attenuated total reflectance Fourier transform-infrared (ATR FT-IR) spectrophotometer (FT-IR Alpha 2, BRUKER). The XPS spectra were obtained with a Nexsa electron spectrometer from Thermo Fisher Scientific (Waltham, MA, USA) using monochromated Al-Kα radiations. The pressure was 2.0 × 10^−8^ mbar. The binding energies were referenced to the C1s line at 284.8 eV from adventitious carbon. The hardness of the films was assessed by the industrial pencil hardness test (ASTM D3363) using Mitsubishi pencils (Tokyo, Japan) of different hardnesses at a load of 100 g. The adhesion of the films was examined by the cross-cut tape test (ASTM D3359) by producing a grid with meshes 0.5 mm × 0.5 mm in size cut with a cross-hatcher. Two types of tapes, a weakly adhesive 3 M scotch tape and a strongly adhesive 3 M VHB tape, were applied to the grid and firmly rubbed onto the film with a finger before being abruptly and vertically peeled off. The grids were then observed through an optical microscope, and the meshes were evaluated for delamination. The atmospheric corrosion resistance was tested by exposing the films to a 5% NaCl solution at 35 °C for 24 h. The haze measurement test was conducted by comparing the transmission of the films before and after the salt test.

## 3. Results and Discussion

**Scheme 1:**
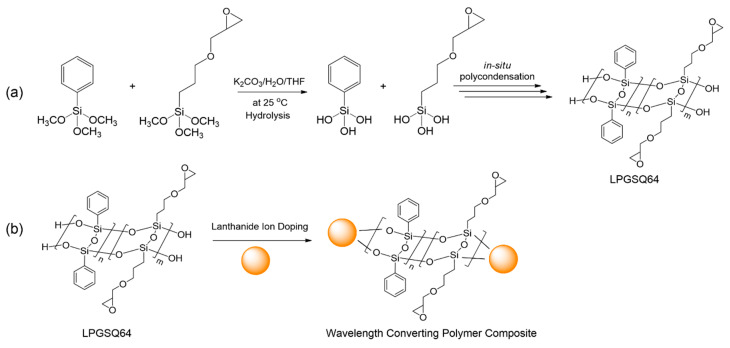
(**a**) Synthesis of the hybrid polymer matrix ladder-structured poly(phenyl-co-glycidoxypropyl) silsesquioxane (LPGSQ64); (**b**) lanthanide ion coordination.

**Figure 1:**
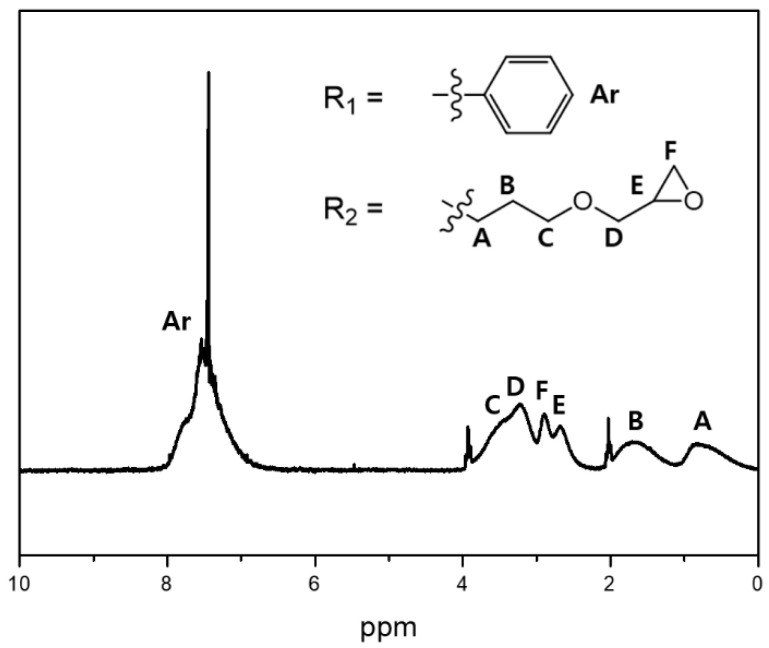
^1^H NMR spectrum of poly(phenyl-co-glycidoxypropyl) silsesquioxane (LSGSQ64).

The synthesis of the ladder-structured poly(phenyl-co-glycidoxypropyl) silsesquioxane (LPGSQ64) followed our literature procedure [30], in which trialkoxysilane monomers were hydrolyzed and underwent in situ polycondensation to give a high molecular weight polymer as shown in Scheme 1a. According to the TGA measurements, M_w_ was 43,000 g mol^−1^ with PDI of 2.4. The organic functional—R groups were selected to provide mechanical hardness and high refractive index for the phenyl group and thermal cross-linking function for the glycidoxypropyl epoxy groups. ^1^H NMR and 29Si NMR are provided in Table 1 and Figure 1.

The obtained LPGSQ64 polymer was then utilized as a polymer matrix to form lanthanide-based composite films with highly compatible interactions between lanthanide ions and the terminal silanol groups of LPGSQ64, as presented in Scheme 1b [31].

**Figure 2:**
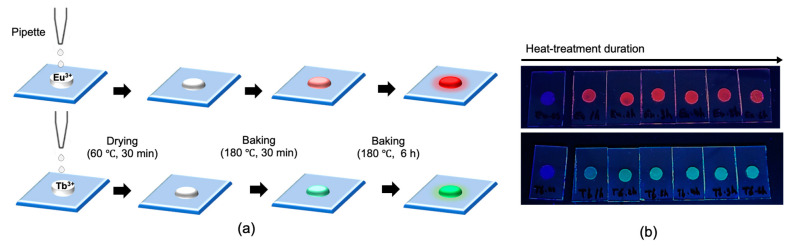
(**a**) Lanthanide LPSQs film fabrication process; (**b**) photographs of the fabricated luminescent films under a UV lamp (λ = 365 nm). Thermal curing duration at 180 °C increases by 1 h from left to right.

To fabricate the luminescent hybrid films, the precursor solutions were first made by mixing a certain amount of lanthanide precursors (Eu^3+^ and Tb^3+^) in a 20 wt% LPGSQ64 in THF solution, then drop-cast onto the glass substrate and kept at room temperature from 5–10 min. Second, 60 °C preheating was applied in ambient air for 30 min to produce a transparent film. Finally, a fluorescent film was obtained after the heat treatment at 180 °C in ambient air for 6 h. Figure 2a presents the film fabrication process, where Eu^3+^ generates red-emission color under UV light irradiation and Tb^3+^ generates green emission, respectively. Figure 2b shows the fabricated films cured at 180 °C for various heat treatment intervals (from 0 to 6 h), illuminated under a UV lamp (λ = 365 nm). The film was fabricated immediately after the solvent evaporation at 60 °C, but it lacked color emission without the additional heat treatment (see the first glass slide in Figure 2b). As the heat treatment time increased from 1 to 6 h, the intensity of the emitted light increased proportionally.

The curing temperature of 180 °C was selected to minimize the unwanted yellowing effect of the transparent film with the temperature increase to 200 °C (Figure S1). While the photoluminescence (PL) intensity of the samples cured at 200 °C increased overall from 30 min to 7 h of curing, UV–vis transmittance was constant only for samples cured for 30 min and 1 h, and then dropped significantly for samples cured longer than 2 h at 200 °C (Figure S1b for Eu^3+^ doping and Figure S1d for Tb^3+^ doping). These results indicate that the films could not resist prolonged heat treatment at 200 °C. Thus, all films discussed in the following text were cured at 180 °C.

**Figure 3:**
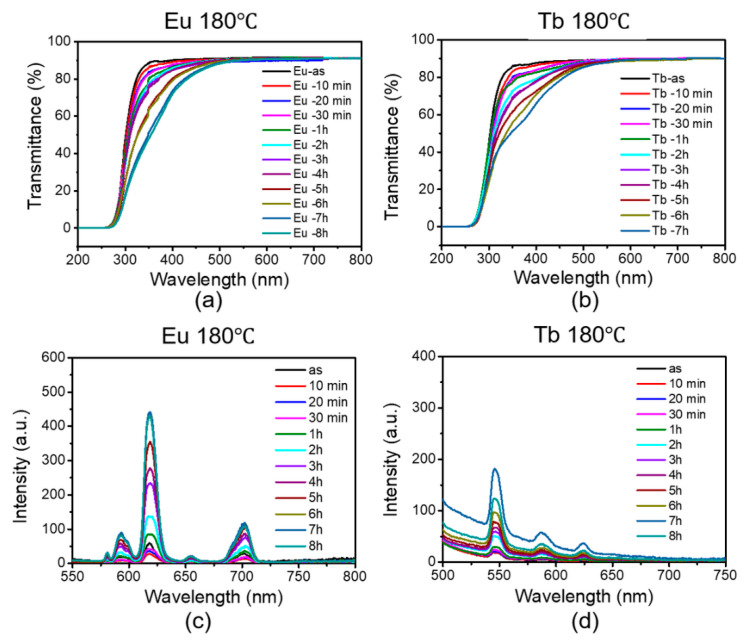
UV–Vis transmittance spectra of (**a**) LPGSQ64−Eu and (**b**) LPGSQ64−Tb films with various heat treatment durations at 180 °C; PL luminescence (excitation at 365 nm) spectra of (**c**) LPGSQ64–Eu and (**d**) LPGSQ64–Tb films with various heat treatment durations at 180 °C.

**Scheme 2:**
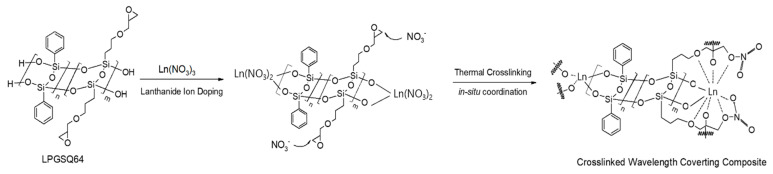
Proposed cross-linking mechanism of the ladder-structured poly(phenyl-co-glycidoxypropyl) silsesquioxane (LPGSQ64) and lanthanide ions during thermal curing.

Interestingly, there was no luminescence from a transparent precursor solution and as-synthesized film under UV light. However, after heat treatment (180 °C, 1 h), a slight yellowing under natural light occurred, while the film showed transmittance above 80% at >350 nm. In contrast, Eu^3+^- and Tb^3+^-doped films exhibited red and green luminescence under UV light, respectively, as shown in Figure 2. The transmittance spectra (Figure 3a,b) indicate that the yellowing phenomenon becomes more severe as the heat treatment was performed over a long period of time, while PL intensity (Figure 3c,d) gradually increased and reached its maximum after 6 h of heat treatment. The increase in both Eu^3+^ and Tb^3+^ PL intensity with heat treatment duration can be attributed to the strengthening of LPGSQ64-Ln cross-linking. Eu^3+^- and Tb^3+^-doped films’ luminescence comes from the lanthanide coordination with LPGSQ64, generating a covalent Si–O–Ln bond. Furthermore, lanthanide nitrates can be bonded to the polymer through a ring-opening reaction of a neighboring epoxy group. This reaction leads to the formation of an alkoxy group, where oxygen becomes electrostatically coordinated with the lanthanide-doped LPGSQ64 complex. Furthermore, additional lanthanide coordination is then expected with the NO_3_^−^ bonded via oxygen to the emerged alkoxy group. This multiple lanthanide–polymer coordination, thus, results in strengthened cross-linking and strong luminescence [32,33]. As a result, prolonged heat treatment promotes the epoxy ring opening reaction, forming more alkoxy groups and providing more coordination sites. This eventually leads to sufficient cross-linking and higher PL intensity values. Scheme 2 shows the possible cross-linking mechanism during the heat treatment. On the other hand, it is hypothesized that the epoxy ring opening reactions can generate other functional groups, such as ketone or ethylene groups [34], which can be color centers leading to the unfavorable yellowing effect. Thus, this hypothesis explains the increase in the yellowing effect with heat treatment duration and concentration (discussed in the next paragraph).

**Figure 4:**
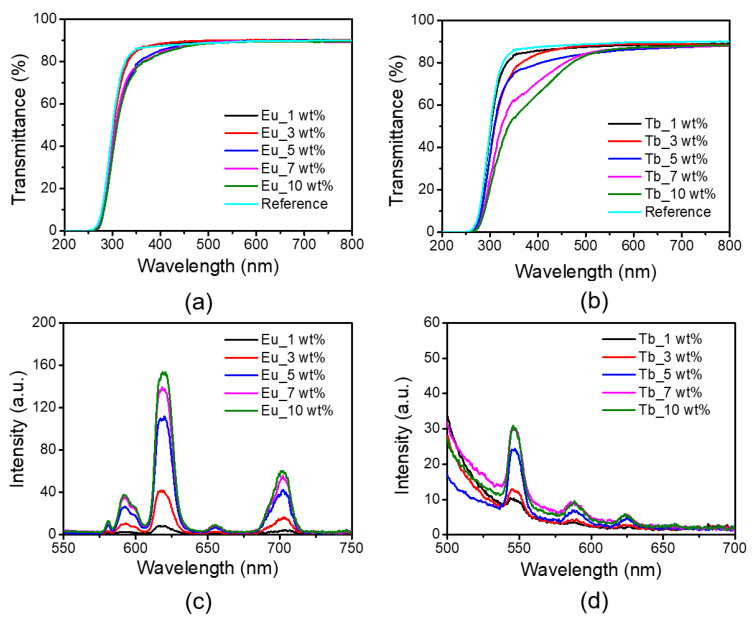
UV–Vis transmittance spectra of (**a**) LPGSQ64–Eu and (**b**) LPGSQ64–Tb films with different doping concentrations; PL luminescence spectra of (**c**) LPGSQ64–Eu and (**d**) LPGSQ64–Tb films with different doping concentrations.

Figure 4 illustrates the UV–vis transmittance and PL spectra of the polymer composite films doped at different concentrations of Eu^3+^ and Tb^3+^. The measured transmittance of the samples with low lanthanide concentration showed high transparency. However, as the concentration was increased, slight yellowing, exhibited as a broad absorption from 300~500 nm, occurred in 5%-doped Eu^3+^ films, which remained unchanged for 7%- and 10%-doped films (Figure 4a). In the case of the Tb^3+^-doped films, yellowing appeared at 3 wt% and kept increasing with the doping level of Tb^3+^ up to 10 wt % (Figure 4b). As shown by the PL spectra (Figure 4c,d), only fluorescence caused by Tb^3+^ and Eu^3+^ was observed, where the intensity of Tb^3+^-doped films was relatively lower than that of Eu^3+^-doped films. However, the photoluminescence (PL) intensity depends on the type of activator (Ln^3+^) and host material (such as LPGSQ64), as well as their interactions. Therefore, a direct comparison of the PL intensities between LPGSQ64–Eu and LPGSQ64–Tb was not possible. The peaks at 570, 585, 612, 660, and 700 nm are the typical emission of Eu^3+^ ion, arising from its ^5^D_0_ excited state to ^7^F_0_, ^7^F_1_, ^7^F_2_, ^7^F_3_, and ^7^F_4_ ground state, respectively (Figure 4c). The peaks at 545, 584, and 620 nm are the typical emission of Tb^3+^ ion, arising from its ^5^D_4_ excited state to ^7^F_5_, ^7^F_4_, and ^7^F_3_ ground state, respectively (Figure 4d) [3,12]. When the concentrations of Eu^3+^ and Tb^3+^ increase, the ^5^D_0_→^7^F_J_ transition of Eu^3+^ and the ^5^D_4_→^7^F_J_ transition of Tb^3+^ are enhanced. Therefore, intensities of both Eu- and Tb-doped films increased with the doping concentration.

The PL spectra of LPGSQ64 films doped with 100% Eu, 100% Tb, and a 50% Eu/50% Tb mixture indicated that energy transfer occurs from Tb^3+^ to Eu^3+^ (Figure S3), consistent with a previous work by Cui et al. [35]. Furthermore, the PL spectra showed that the intensity of Tb^3+^ emission in the LPGSQ64–Tb film decreased by approximately 88% in the LPGSQ64–Eu:Tb (1:1) film, whereas the intensity of Eu^3+^ emission in the LPGSQ64–Eu film decreased by only 46% in the LPGSQ64–Eu:Tb (1:1) film. This difference in the PL intensity reduction is due to the energy transfer from Tb to Eu.

**Figure 5:**
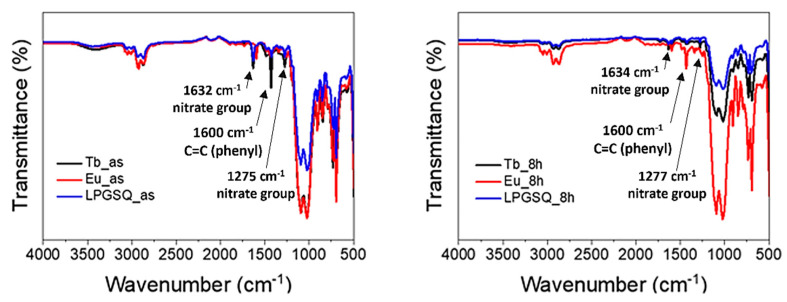
FT−IR spectra of as-prepared (**left**) and 8 h−cured LPGSQ64, LPGSQ64–Eu, and LPGSQ64–Tb films (**right**). The arrows demonstrate the newly emerged peaks identified in the LPGSQ film after lanthanide doping (nitrate groups) from 1632–1634 cm^−1^ and 1275−1277 cm^−1^; C=C bonds from phenyl groups present in both undoped and doped films from 1590–1600 cm^−1^.

The undoped and lanthanide-doped LPGSQ64 films were analyzed by FT-IR spectroscopy before and after curing at 180 °C for 8 h. The as-prepared films with dopants showed absorption peaks not native to the LPGSQ64 film at the wavenumbers from 1632–1634 and 1275–1277 cm^−1^ (Figure 5), which correspond to the nitrate stretching modes [36]. The peaks from 1590–1600 cm^−1^, interpreted as C=C bonds from the phenyl group [7], were observed in both undoped and doped films, indicating the inclusion of the phenyl group in the LPGSQ polymer. When cured films were compared, it was seen that the intensity of the phenyl peak at 1600 cm^−1^ decreased. When exposed to elevated temperatures, phenyl groups can undergo decomposition to benzyne, acetylene, and diacetylene groups [37], becoming color centers and inducing the unwanted yellowing effect discussed previously.

**Figure 6:**
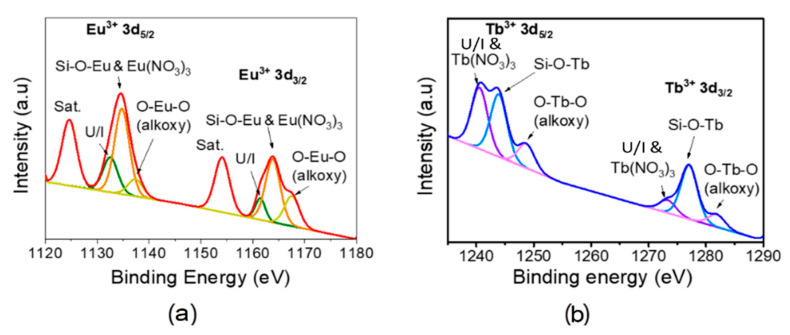
(**a**) High-resolution deconvoluted peaks for Eu^3+^ 3d of LPGSQ64–Eu, (**b**) high-resolution deconvoluted peaks for Tb^3+^ 3d of LPGSQ64–Tb.

Furthermore, XPS measurements were conducted in order to estimate how many metal centers were involved in different types of lanthanide–polymer coordination suggested in Scheme 2. Wide-scan XPS spectra of the undoped LPGSQ64, LPGSQ64–Eu, and LPGSQ64–Tb confirm the occurred coordination between the polymer and the lanthanides (Figure S4). The positions of deconvoluted peaks in high-resolution spectra shown in Figure 6a,b were studied in detail and compared to the previously reported XPS data to fully understand the polymer–lanthanide coordination. The deconvoluted spectra of Eu^3+^ 3d (Figure 6a) are assigned to two peaks at 1134 and 1137 eV for Eu^3+^ 3d_5/2_ and 1164 and 1167 eV for Eu^3+^ 3d_3/2_. The remaining peaks at 1131 and 1161 eV were left unassigned due to the absence of relevant references and were believed to emerge from impurities present in the system, given their low intensity. Since the primary type of the coordination suggested for the lanthanide–polymer matrix was via the Si–O bond, the peaks with the highest intensity at 1134 and 1164 eV were assigned to the Si–O–Eu coordination, which corresponded to the Eu^3+^ 3d binding energies of the reported SiO_2_:Eu^3+^ complex [38]. The peaks at 1137 and 1167 eV were assigned to the second type of the suggested coordination via O–Eu–O, involving the alkoxy groups that emerged from the epoxy ring opening reaction [39]. The binding energy of Eu–NO_3_ is reported at 1136.4 eV, which overlaps with the Si–O–Eu peak at 1134 eV [40]. Thus, it hindered the possibility of comparing how many lanthanide centers were involved in the Si–O–Eu coordination and how many remained uncoordinated.

On the other hand, in the deconvoluted spectra of Tb^3+^ 3d, it was possible to partially separate Si–O–Tb and Tb–NO_3_ peaks. The peaks at 1240 and 1273 eV were assigned to Tb^3+^ 3d_5/2_ and Tb^3+^ 3d_3/2_ of Tb–NO_3_, respectively [41]. The peaks of Si–O–Tb coordination were identified at 1243 and 1276 eV [42]. By comparing the intensities of these peaks, it can be estimated that an equal amount of lanthanide centers is involved in the coordination with the polymer matrix via Si–O and remains uncoordinated as Tb(NO_3_)_3_. Lastly, the peaks at 1248 and 1281 eV were assigned to the O–Tb–O coordination, suggesting the Tb^3+^ coordination with the O-containing alkoxy group [43].

**Figure 7:**
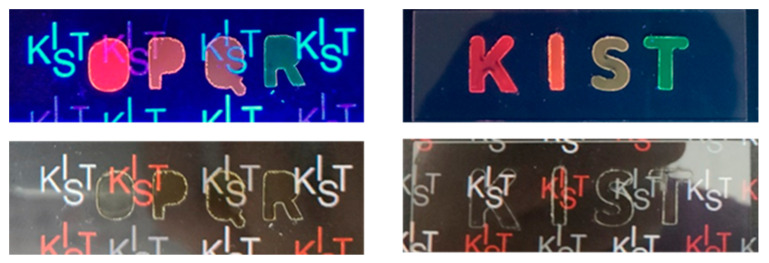
Alphabet-shaped “K-I-S-T” thin films on a slide glass prepared by varying the lanthanide doping ratio. Photographs were taken under 365 nm UV light irradiation (**top**) and under natural light (**bottom**).

Figure 7 demonstrates images of differently colored fluorescent films prepared by varying the ratio of lanthanide doping and photographed under both natural and UV light (λ = 365 nm). The films were made in the shape of alphabetic stickers, “O-P-Q-R” and “K-I-S-T”, on a slide glass, respectively. The ratio of Eu^3+^ and Tb^3+^ doping was controlled according to the preliminary experiments to produce the desired color of the fluorescent films (Figure S2). To prepare orange and yellow, 5 wt% of Eu^3+^- and 7 wt% of Tb^3+^-containing solution were mixed in a 1:3~1:7 ratio, respectively. The “O-P-Q-R” and “K-I-S-T” alphabet-shaped thin films show that the desired color variation was effectively achieved, where Eu^3+^ (‘O’ and ‘K’) under the 365 nm UV excitation gives red, Tb^3+^ (‘R’ and ‘T’) gives green, Eu^3+^_1_:Tb^3+^_3_ (‘P’ and ‘I’) gives orange, Eu^3+^_1_:Tb^3+^_5_ (‘Q’) gives orange yellow, and Eu^3+^_1_:Tb^3+^_7_ (‘S’) gives yellow.

Finally, wear resistance tests, including pencil hardness, cross-cut adhesion, and environmental corrosion, were performed to evaluate the suitability of the lanthanide-doped LPGSQ64 films for practical applications. The pencil hardness was estimated using a pencil hardness tester by the standard protocol (ASTM D3363). A pencil was positioned above the samples, and the angle between the pencil and the sample was set to 45°. Table 2 shows that the hardness of the samples cured at 180 °C for 6 h was significantly higher than that of the samples cured for 1 h. Thus, it is evidenced that full cross-linking occurs within the lanthanide–polymer hybrid when the films are thermally cured for 6 h. It can be explained by the strengthened coordination of lanthanide atoms via Si–O–Ln and alkoxy and nitrate groups to lanthanide atoms via the epoxy ring opening reaction. Furthermore, Table 1 demonstrates that the hardness of the undoped LPGSQ films cured for 1 h and 6 h was low, not greater than HB, which would pose a major obstacle for their practical applications. In contrast, both Eu^3+^- and Tb^3+^-doped films exhibited a significant improvement in the films’ hardness. Moreover, the hardness of the doped films was improved as the doping percentage increased, which resulted from the formation of more coordination sites, which strengthened the cross-linking within the lanthanide–polymer composite.

The adhesion of the undoped and lanthanide-doped LPGSQ64 films was also evaluated by a standard protocol (ASTM D3359), employing two types of tapes with various adhesive strengths. For LPGSQ64 films cured for 1 h and 6 h, partial delamination and fragment separation were seen along the prefabricated scratches, indicating insufficient film adhesion. In comparison, no visible delamination was observed for Eu^3+^- and Tb^3+^-doped LPGSQ films even after repeating the cross-cut tape tests ten times (Figure S4), demonstrating the superior adhesion properties provided by the lanthanide doping. Lastly, the behavior of the films in a saline environment was tested with regards to atmospheric corrosion by exposing the films to a 5% NaCl solution at 35 °C for 24 h. The haze test was then performed by comparing the transmission of the films before and after the salt test. Figure S5 demonstrates no discernible drop in the transmittance after the salt test, indicating resistance to atmospheric corrosion. Thus, it is believed that the lanthanide-doped highly transparent LPGSQ64 films with their advantageous wear resistance properties can be utilized for practical applications, such as color conversion displays.

## 4. Conclusions

In this study, a ladder-like polysilsesquioxane (LPGSQ64) polymer was synthesized by a facile method and effectively doped with lanthanide metals, such as europium and terbium, to fabricate highly transparent fluorescent films. Various emissive colors of the fluorescent films were successfully produced by controlling the appropriate amount of the lanthanides, demonstrating the wide range of color emission for the lanthanide-doped LPGSQ64 films. For instance, red and green colors were obtained from Eu- and Tb-LPGSQ64 films, while Eu_1_:Tb_3_–, Eu_1_:Tb_5_–, and Eu_1_:Tb_7_–LPGSQ64 films resulted in orange, orange yellow, and yellow colors, respectively.

The optical properties of films were investigated through transmittance, PL and FT-IR measurements to understand the nature of fluorescence appearance after lanthanide doping and subsequent heat treatment. It was determined that, for the lanthanide-doped LPGSQ64 polymer, the optimal curing temperature was 180 °C. In contrast, the elevation to higher temperatures led to an unwanted yellowing phenomenon that hindered transparency and photoluminescent properties. Furthermore, PL spectra suggested that the increase in doping concentration and duration of heating treatment increased the PL intensity, which is attributed to the increased rate of the ^5^D_0_→^7^F_J_ transition of Eu^3+^ and the ^5^D_4_→^7^F_J_ transition of Tb_3+_.

XPS analysis was performed to study the proposed mechanism of the lanthanides coordination with the LPGSQ64 polymer, which was hypothesized to combine the primary Ln coordination via Si–O bonds of the polymer matrix; the O–Ln–O coordination via the alkoxy group emerging from the epoxy ring opening reaction with lanthanide nitrates; the Ln–O–N coordination of the nitrate group bonded to the neighboring alkoxide. This 9-fold coordination led to the strengthened cross-linking with the polymer–lanthanide matrix. Thus, it increased photoluminescence and enhanced the films’ wear resistance by improving their hardness, adhesion, and atmospheric resistance to a saline environment. In conclusion, it is believed that the proposed highly transparent luminescent lanthanide-doped LPGSQ64 films with high wear resistance and effective color tuning properties can be suitable candidates for color conversion layers in commercial display fabrication.

## Figures and Tables

**Table 1:** Assignments of ^1^H and ^29^Si NMR for the ladder-structured poly(phenyl-co-glycidoxypropyl) silsesquioxane (LSGSQ64).

Formula	Chemical Shift (ppm)
^1^H NMR	
t, Si(C***H***_2_CH_2_CH_2_OCH_2_CHOCH_2_), 2H	0.35–0.75
m, Si(CH_2_C***H***_2_CH_2_OCH_2_CHOCH_2_), 2H	1.3–1.7
d, Si(CH_2_CH_2_CH_2_OCH_2_C***H***OCH_2_), 1H	2.5–2.7
m, Si(CH_2_CH_2_CH_2_OCH_2_CHOC***H***_2_), 2H	2.8–3.0
m, Si(CH_2_CH_2_CH_2_OC***H***_2_CHOCH_2_), 2H	3.0–3.3
m, Si(CH_2_CH_2_C***H***_2_OCH_2_CHOCH_2_), 2H	3.2–3.4
m, Si(C_6_***H***_5_), 5H	7.2–8.0
^29^Si NMR	
***Si***(CH_2_CH_2_CH_2_OCH_2_CHOCH_2_)	−64 to −70
***Si***(C_6_H_5_)	−77 to −82

**Table 2:** Pencil hardness and delamination fraction (adhesion) from a cross-cut tape test of the LPGSQ64, LPGSQ64–Eu, and LPGSQ64–Tb films cured for 1 h and 6 h with 3% and 5% doping.

Sample	Pencil Hardness (10H = Best)	Cross-Cut Adhesion (5B = Best)
1 h curing		
LPGSQ64	2B	3B
LPGSQ64-Eu 3%	2H	5B
LPGSQ64-Tb 3%	HB	5B
LPGSQ64-Eu 5%	3H	4B
LPGSQ64-Tb 5%	H	5B
6 h curing		
LPGSQ64	HB	3B
LPGSQ64-Eu 3%	3H	5B
LPGSQ64-Tb 3%	H	5B
LPGSQ64-Eu 5%	3H	5B
LPGSQ64-Tb 5%	4H	5B

## Data Availability

Data are contained within the article.

## Supplementary Materials

The following supporting information can be downloaded at: https://www.mdpi.com/article/10.3390/ma16062537/s1, Figure S1: PL luminescence and transmittance of LPGSQ64-Eu and LPGSQ64-Tb films cured at 200℃ with different time durations (30 min–7 h); Figure S2: PL spectra of (a) LPGSQ64-Eu, (b) LPGSQ64-Tb, (c) LPGSQ64-Eu:Tb (1:3), and (d) LPGSQ64-Eu:Tb (1:5) cured at 180 ℃ for 6 h, (e) circular thin films on a slide glass prepared by LPGSQ64-Eu, LPGSQ64-Eu:Tb (1:3), LPGSQ64-Eu:Tb (1:5), and LPGSQ64-Tb, from left to right. Photographs were taken under 365 nm UV light irradiation (left) and under natural light (right); Figure S3: Superposed PL spectra of (a) LPGSQ64-Eu:Tb (1:1), (b) LPGSQ64-Eu, and (c) LPGSQ64-Tb are shown. The green arrow indicates an 88% reduction in the intensity of green luminescence, while the red arrow indicates a 46% reduction in the intensity of red luminescence; Figure S4: Wide-scan XPS spectra of LPGSQ64, LPGSQ64-Eu, and LPGSQ64-Tb; Figure S5: Microscope images of the LPGSQ64, LPGSQ64-Eu films, and LPGSQ64-Tb films cured for 1 h and 6 h with 3% and 5% doping after the cross-cut tape test. Additionally, images of the films cured for 6 h cut by a cross-hatcher before undergoing the adhesion test are included; Figure S6: Transmittance of LPGSQ64, LPGSQ64-Eu, and LPGSQ64-Tb films with 3% and 5% doping before and after the salt test.
