# Supplementary material for: Lanthanide and Ladder-Structured Polysilsesquioxane Composites for Transparent Color Conversion Layers
Source: Materials (Basel). 2023 Mar 22;16(6):2537. doi: 10.3390/ma16062537 (PMC10053414; doi:10.3390/ma16062537)
Supplement: Supplementary file 1 [file materials-16-02537-s001.zip › materials-2193903-supplementary.pdf]

Supporting Information

Lanthanide and Ladder-Structured Polysilsesquioxane Composites for Transparent Color Conversion Layers

Jaehyun Han [1,2,†], Darya Burak [1,3,†], Valeriia Poliukhova [1], Albert S. Lee [1], Hoseong Jang [1,3], Seungsang Hwang [1,3], Kyung-Youl Baek [1,3], Joonsoo Han [1], Byeong-Kwon Ju [2,*] and So-Hye Cho [1,3,*]

1 Materials Architecturing Research Center, Korea Institute of Science and Technology, 5 Hwarang-ro 14-gil, Seongbuk-gu, Seoul 02792, Republic of Korea
2 Display and Nanosystem Laboratory, College of Engineering, Korea University, Seoul 02841, Republic of Korea
3 Department of Nanomaterial Science and Engineering, Korea University of Science and Technology,
217 Gajeong-ro, Yuseong-gu, Daejeon 34113, Republic of Korea
* Correspondence: bkju@korea.ac.kr (B.-K.J.); sohyec@kist.re.kr (S.-H.C.)
† These authors contributed equally to this work.

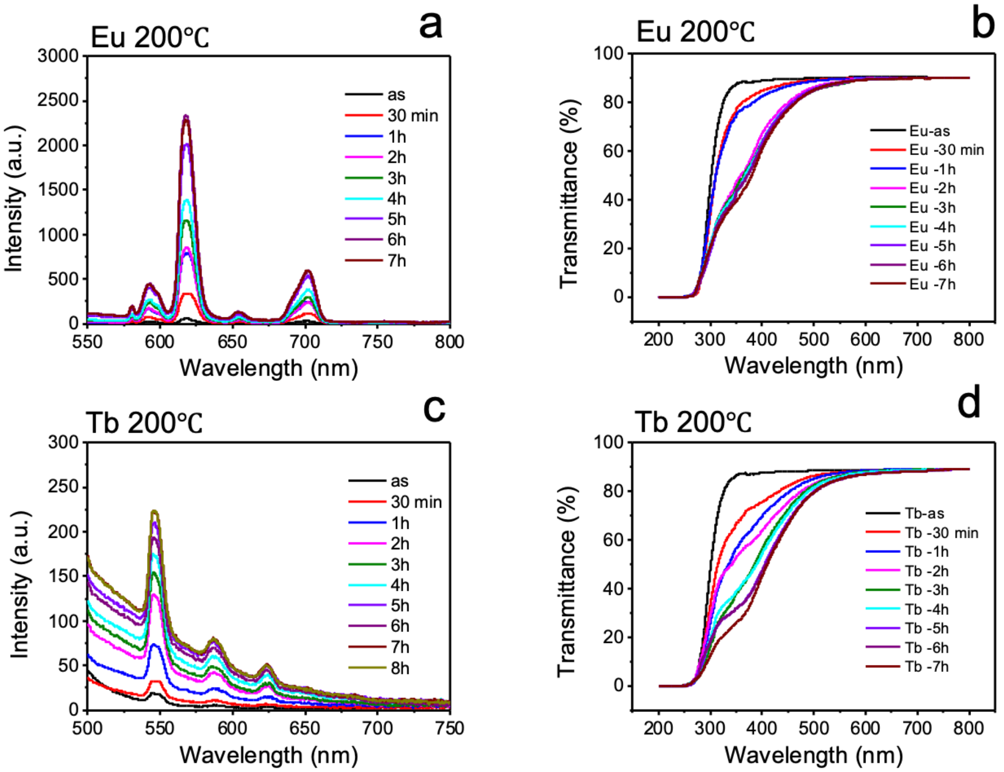

Figure S1. (**a**) PL luminescence and (**b**) transmittance of LPGSQ64-Eu films cured at 200℃ with different time duration (30 min – 7 hours); (**c**) PL luminescence and (**d**) transmittance of LPGSQ64-Tb films cured at 200℃ with different time duration (30 min – 7 hours).

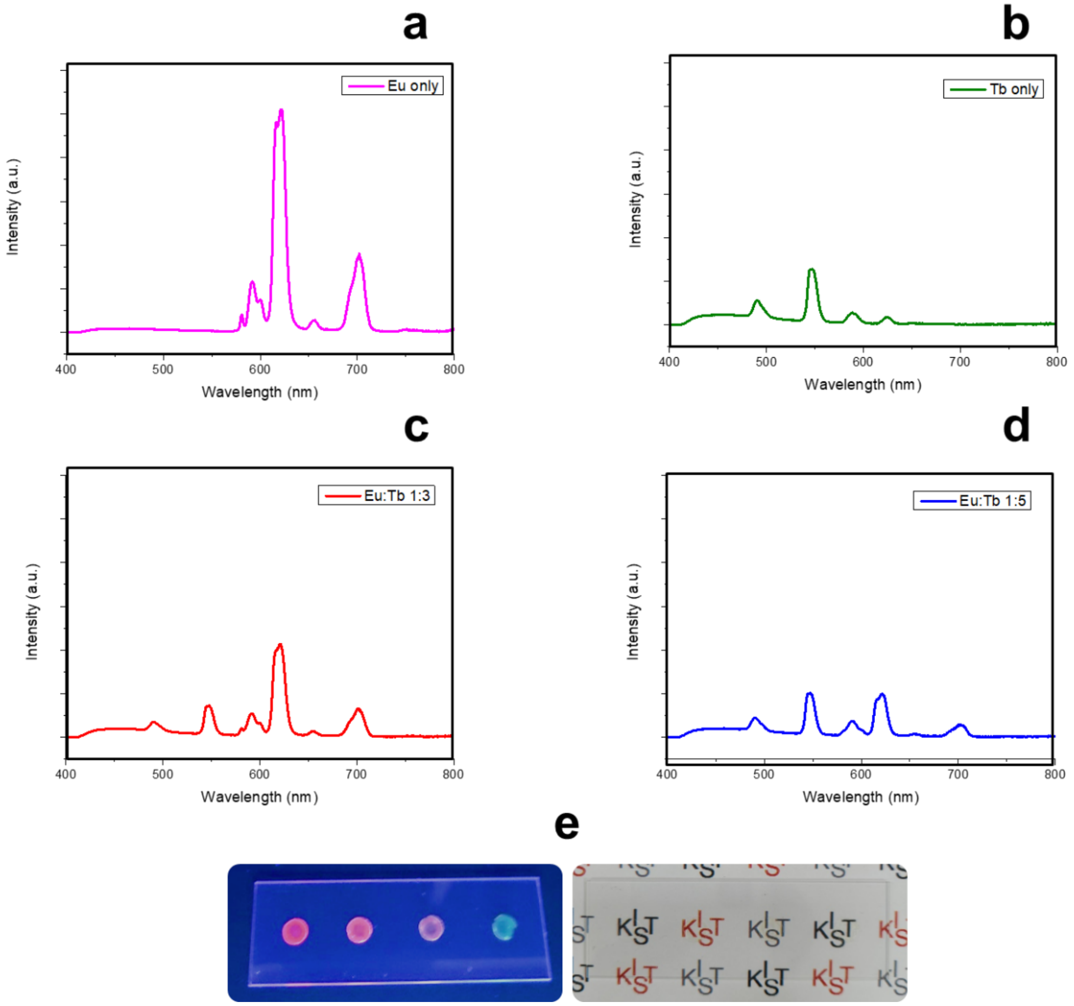

Figure S2. PL spectra of (a) LPGSQ64-Eu, (b) LPGSQ64-Tb, (c) LPGSQ64-Eu:Tb(1:3), and (d) LPGSQ64-Eu:Tb(1:5), cured at 180℃ for 6 h, (e) circular thin films on a slide glass prepared by LPGSQ64-Eu, LPGSQ64-Eu:Tb(1:3), and LPGSQ64-Eu:Tb(1:5), and LPGSQ64-Tb, from left right. Photographs were taken under 365 nm UV light irradiation (left) and under natural light (right).

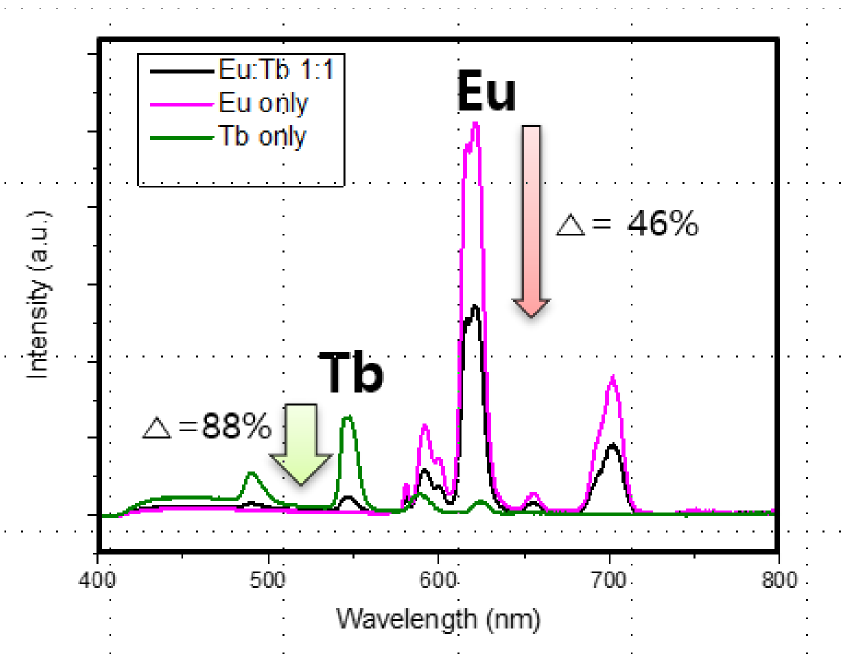

Figure S3. Superposed PL spectra of (a) LPGSQ64-Eu:Tb(1:1), (b) LPGSQ64-Eu, and (c) LPGSQ64-Tb are shown. The green arrow indicates an 88% reduction in the intensity of green luminescence, while the red arrow indicates a 46% reduction in the intensity of red luminescence.

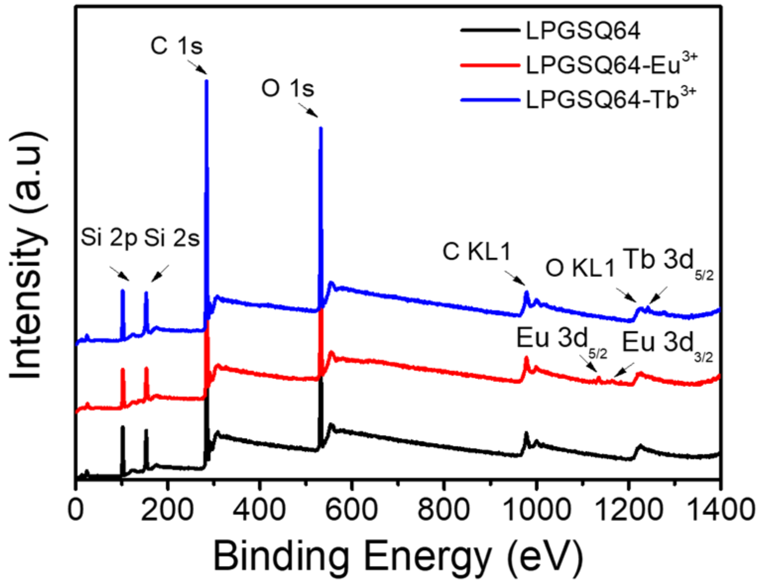

Figure S4. Wide-scan XPS spectra of LPGSQ64, LPGSQ64-Eu, and LPGSQ64-Tb.

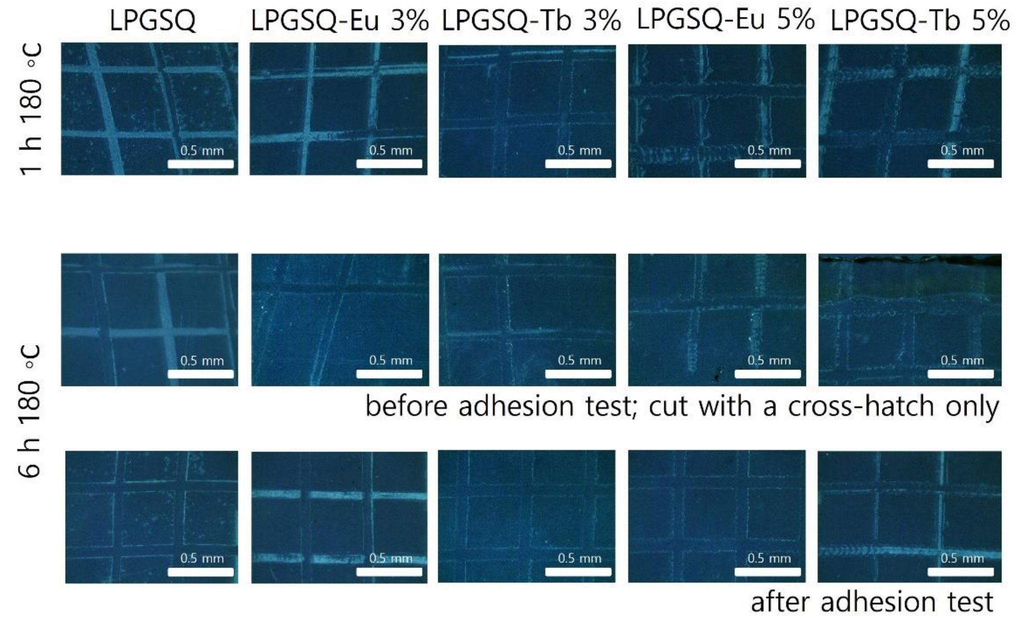

Figure S5. Microscope images of the 1 h and 6 h cured LPGSQ64, LPGSQ64-Eu films, and LPGSQ64-Tb films with 3% and 5% doping after the cross-cut tape test. Additionally, images of the 6 h cured films cut by a cross-hatcher before undergoing the adhesion test are included.

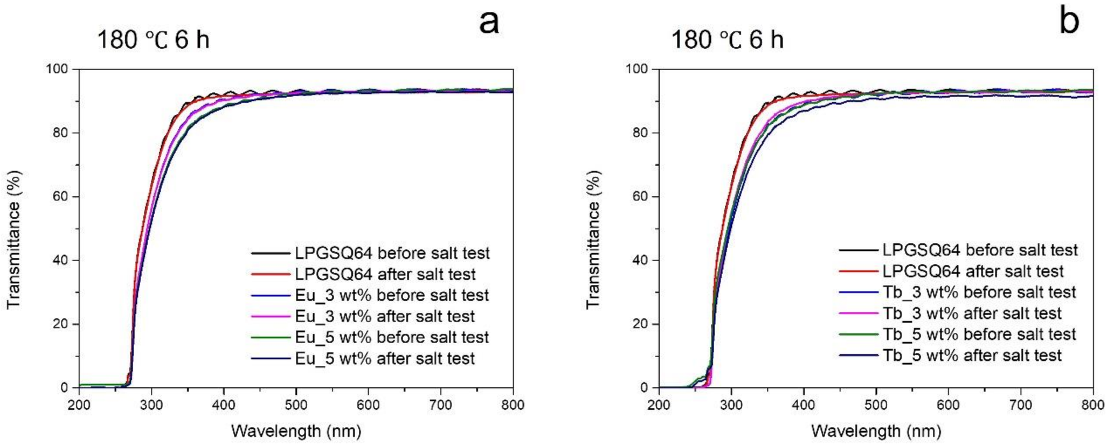

Figure S6. Transmittance of (a) LPGSQ64 and LPGSQ64-Eu films, and (b) LPGSQ64 and LPGSQ64-Tb films with 3% and 5% doping before and after the salt test.
